# Benzoxaborole treatment perturbs S-adenosyl-L-methionine metabolism in *Trypanosoma brucei*

**DOI:** 10.1371/journal.pntd.0006450

**Published:** 2018-05-14

**Authors:** Pieter C. Steketee, Isabel M. Vincent, Fiona Achcar, Federica Giordani, Dong-Hyun Kim, Darren J. Creek, Yvonne Freund, Robert Jacobs, Kevin Rattigan, David Horn, Mark C. Field, Annette MacLeod, Michael P. Barrett

**Affiliations:** 1 Wellcome Centre for Molecular Parasitology, Institute of Infection, Immunity and Inflammation, College of Medical, Veterinary and Life Sciences, University of Glasgow, Glasgow, United Kingdom; 2 Centre for Analytical Bioscience, Division of Molecular and Cellular Sciences, School of Pharmacy, The University of Nottingham, Nottingham, United Kingdom; 3 Department of Biochemistry and Molecular Biology, Drug Delivery Disposition and Dynamics, Monash Institute of Pharmaceutical Sciences, Monash University, Parkville, Victoria, Australia; 4 Anacor Pharmaceuticals, Inc., Palo Alto, California, United States of America; 5 Wellcome Centre for Anti-Infectives Research, School of Life Sciences, University of Dundee, Dundee, United Kingdom; McGill University, CANADA

## Abstract

The parasitic protozoan *Trypanosoma brucei* causes Human African Trypanosomiasis and Nagana in other mammals. These diseases present a major socio-economic burden to large areas of sub-Saharan Africa. Current therapies involve complex and toxic regimens, which can lead to fatal side-effects. In addition, there is emerging evidence for drug resistance. AN5568 (SCYX-7158) is a novel benzoxaborole class compound that has been selected as a lead compound for the treatment of HAT, and has demonstrated effective clearance of both early and late stage trypanosomiasis *in vivo*. The compound is currently awaiting phase III clinical trials and could lead to a novel oral therapeutic for the treatment of HAT. However, the mode of action of AN5568 in *T*. *brucei* is unknown. This study aimed to investigate the mode of action of AN5568 against *T*. *brucei*, using a combination of molecular and metabolomics-based approaches.Treatment of blood-stage trypanosomes with AN5568 led to significant perturbations in parasite metabolism. In particular, elevated levels of metabolites involved in the metabolism of S-adenosyl-L-methionine, an essential methyl group donor, were found. Further comparative metabolomic analyses using an S-adenosyl-L-methionine-dependent methyltransferase inhibitor, sinefungin, showed the presence of several striking metabolic phenotypes common to both treatments. Furthermore, several metabolic changes in AN5568 treated parasites resemble those invoked in cells treated with a strong reducing agent, dithiothreitol, suggesting redox imbalances could be involved in the killing mechanism.

## Author summary

Human African Trypanosomiasis (HAT) is a neglected tropical disease affecting mostly rural communities of sub-Saharan Africa. New drugs for this parasitic disease have been slow to develop, but one new class of drug, the benzoxaboroles, has proven to be highly effective in preclinical models and holds promise for on-going clinical trials. The lead compound in this class is AN5568, also known as SCYX-7158. Here, we study the effects of AN5568 on intracellular metabolism of the parasite and show changes to pathways involved in S-adenosyl-methionine metabolism. Further work shows similarities between this drug and sinefungin, an inhibitor of methyltransferases and dithiothreitol, an endoplasmic reticulum stress inducer. These phenotypes provide new clues to the mechanism of action of this compound.

## Introduction

The monoflagellate protozoan parasite *Trypanosoma brucei* is the causative agent of Human African trypanosomiasis (HAT), and is one of three species that cause Nagana in livestock [1]. HAT is prevalent in sub-Saharan Africa and is responsible for a significant socio-economic burden. The majority of HAT cases are caused by *T*. *b*. *gambiense*, in West Africa, with the remaining cases attributed to *T*. *b*. *rhodesiense*, in the east [2]. Cases have decreased in recent years from 38,000 in 1998 to fewer than 3,000 in 2015 [3], leading to the gambiense form of the disease being targeted by the WHO for elimination [4]. The disease is characterised by an early stage infection of the mammalian bloodstream and other tissues, followed by a late stage where the parasite penetrates the blood-brain barrier to proliferate within the central nervous system [1, 5].

Current therapeutics against HAT are inadequate, and in some cases, highly toxic, leading to fatal side effects—including a reactive encephalopathy in significant numbers of patient treated with melarsoprol [6]. There is evidence of resistance to these drugs in the field [7] and current therapeutics frequently target only one *T*. *brucei* subspecies, or either early- or late-stage HAT [8]. Thus, there is a desperate need for novel and improved therapeutics to combat HAT.

An emerging class of boron-containing compounds known as benzoxaboroles have shown promise as therapies against a wide range of diseases including, but not limited to those caused by, viral [9], fungal [10], bacterial [11, 12] and parasitic [13] infections and inflammation [14]. These compounds have been reported to act as inhibitors of kinases [15], tRNA synthetases [16, 17], CPSF3 [18, 19], phosphodiesterases [20, 21], and carbonic anhydrases [22].

The benzoxaborole AN5568 (SCYX-7158) was previously identified as a potent trypanocide from a library screen of benzoxaborole 6-carboxamides [23]. *In vivo* analysis in murine models showed brain exposure was high, with a maximum serum concentration (C_max_) of 10 μg/mL and AUC_0-24 hr_ higher than 100 μg*h/mL, indicating favourable pharmacokinetics for a stage 2 HAT therapeutic [24]. Importantly, the compound can be administered orally and CNS concentrations are maintained above minimum inhibitory concentration (MIC) for at least 20 hours, sufficient for a twice daily dose [24]. The same study observed a 100% cure rate after a regimen lasting 3 days or more, clearing both *T*. *b*. *gambiense* and *T*. *b*. *rhodesiense* infections [24]. The compound started Phase II/III clinical trials in the last quarter of 2016. In summary, AN5568 presents an exciting new therapeutic for the treatment of both early- and late-stage HAT.

Whilst the pharmacokinetic parameters of the benzoxaborole are well understood, little work has been carried out with regard to understanding the mode of action (MoA) of AN5568, aside from one recent study where proteins potentially binding the drug were identified through a chemoproteomics analysis and mutations in selected resistant lines were identified [25]. Indeed, few MoAs have been resolved to date for preclinical benzoxaboroles [16]. Firstly, the anti-fungal Tavaborole, which targets the leucyl-tRNA synthetase, and more recently, an antimalarial that targets the cleavage and polyadenylation specificity factor subunit 3 (CPSF3) [19]. In addition, Crisaborole, a novel compound developed for the treatment of mild to moderate atopic dermatitis, was shown to inhibit phosphodiesterase 4 (PDE4) [21].

An understanding of AN5568 MoA is crucial to enable the identification of the specific drug target, development of further lead compounds, to assist in the choice of partner drugs in combination therapies and to elucidate potential mechanisms of resistance.

Metabolomics, the study of all small molecules, or metabolites, in a given system, offers the possibility of direct drug target identification when drugs inhibit specific enzymes [26, 27]. In an ideal setting, drug inhibition of metabolic enzymes will result in elevated levels of the enzyme’s substrate, with a corresponding reduction in the product [26–28]. In this study, we utilised a liquid chromatography-mass spectrometry (LC-MS) platform, with the aim of elucidating the MoA of AN5568, using the lab-adapted Lister 427 strain of *T*. *b*. *brucei*. Liquid-chromatography mass spectrometry analysis showed significant perturbations in methionine metabolism in drug-treated cells. Further investigation showed that the benzoxaborole is significantly antagonised in the presence of sinefungin, a non-specific methyltransferase inhibitor. In addition, parasites treated with sinefungin and the endoplasmic reticulum stress inducer dithiothreitol (DTT) both bear similar metabolic phenotypes to those observed following treatment with AN5568.

## Results

### *In vitro* activity of AN5568

Efficacy of AN5568 was determined in both bloodstream form (BSF) and procyclic form (PCF) parasites of the Lister 427 strain, by calculating the half maximal effective concentration (EC_50_) (Table 1). In BSFs, mean EC_50_ was 193 ± 48 nM, whilst in contrast, the EC_50_ in PCF parasites was almost 10-fold higher. This suggests some difference in benzoxaborole targeting of the two developmental forms.

**Table 1:** EC_50_ concentrations calculated by alamar Blue for several trypanosomatids.

Organism	EC_50_
***T*. *b*. *brucei*–BSF**	193 ± 48 nM
***T*. *b*. *brucei*–PCF**	1,500 ± 90 nM
***T*. *congolense*–BSF**	509 ± 18 nM
***L*. *mexicana*—promastigote**	37.7 ± 3.6 μM

Further analyses on other trypanosomatids were carried out (Table 1). The EC_50_ in *Trypanosoma congolense*, a closely-related trypanosome species of veterinary importance, was 509 ± 18 nM. In contrast, the promastigote stage of *Leishmania mexicana*, one of the trypanosomatid species responsible for Leishmaniasis, exhibited an EC_50_ of 37.7 ± 3.6 μM, almost 200-fold and 25-fold higher than in *T*. *b*. *brucei* BSF and PCF respectively (Table 1).

### AN5568 provokes profound changes in S-adenosyl-L-methionine levels in *T*. *b*. *brucei*

**Fig 1:**
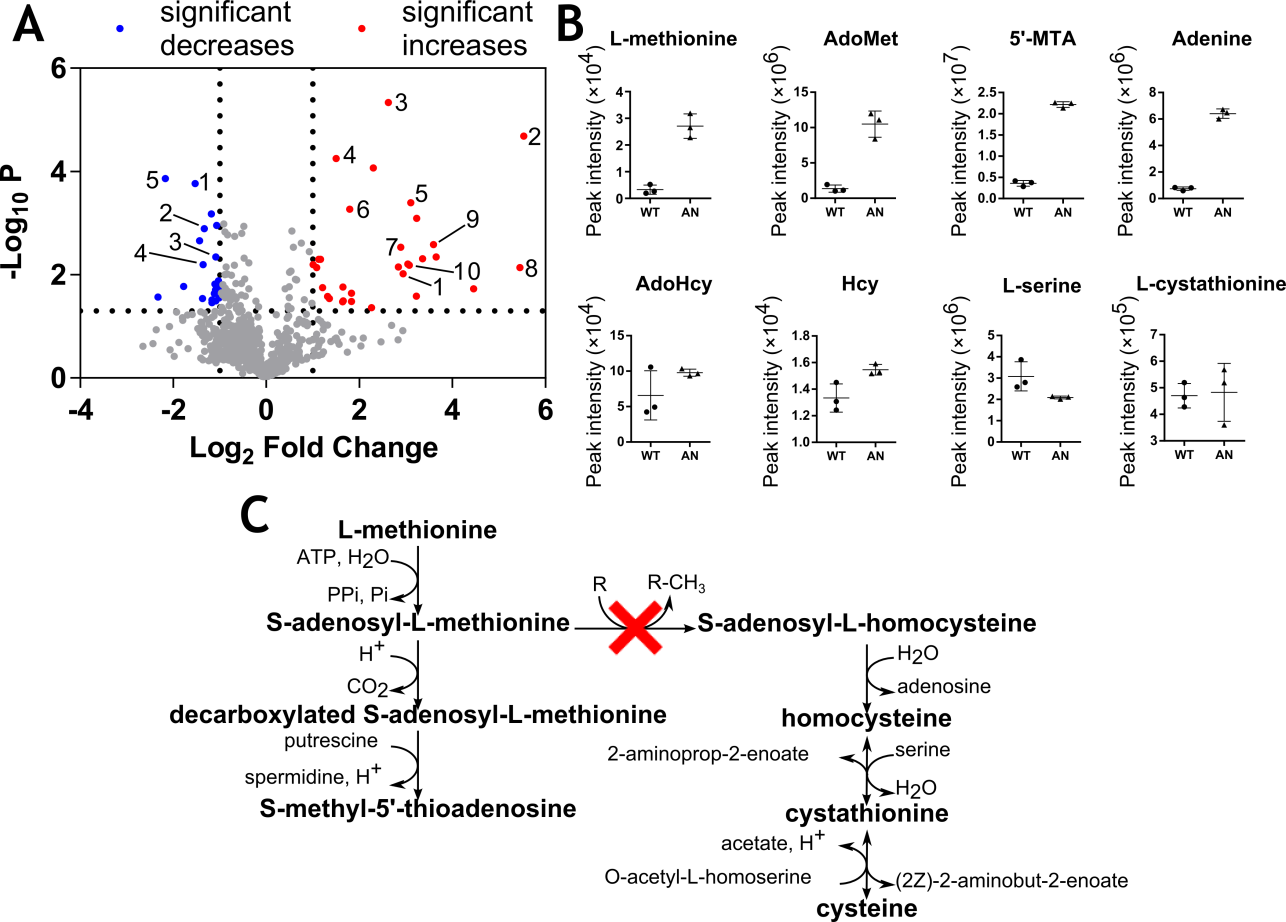
Metabolic changes associated with AN5568 treatment. A) A volcano plot showing the global metabolic changes associated with AN5568 treatment. Numbers at red dots correspond to the following metabolites that were significantly increased **1)** S-adenosyl-L-methionine (m/z: 398.1374, RT: 18.02 min, 7.67-fold), **2)** 1,2-Dihydroxy-5-(methylthio)pent-1-en-3-one (m/z: 162.0350, RT: 17.24 min, 46.33-fold), **3)** 5’-methylthioadenosine (m/z: 297.0896, RT: 7.75 min, 6.17-fold), **4)** N6-acetyl-L-lysine (m/z: 188.1162, RT: 14.70 min, 2.83-fold), **5)** adenine (m/z: 135.0546, RT: 7.71 min, 12.55-fold), 6) 4-hydroxy-4-methylglutamate (m/z: 177.0637, RT: 15.3 min, 3.46-fold), 7) N6,N6,N6-trimethyl-L-lysine (m/z: 188.1524, RT: 23.54 min, 7.40-fold), 8) cyclic ADP-ribose (m/z: 541.0608, RT: 16.90 min, 43.78-fold), 9) aminoacetone (m/z: 73.0528, RT: 7.67 min, 12.06-fold), 10) 8-amino-7-oxononanoate (m/z: 187.1208, RT: 13.75 min, 8.43-fold). Numbers at blue dots correspond to the following metabolites that were significantly decreased: 1) [PC(14:0)] 1-tetradecanoyl-sn-glycero-3-phosphocholine (m/z: 467.3014, RT: 4.75 min, 0.35-fold), 2) sn-glycerol 3-phosphate (m/z: 172.0136, RT: 16.44 min, 0.40-fold), 3) D-glucosamine 6-phosphate (m/z: 259.0457, RT: 17.68 min, 0.47-fold), 4) 2-deoxy-D-ribose 5-phosphate (m/z: 214.0242, RT: 16.45 min, 0.39-fold), 5) Asp-Asp-Cys-Pro (peptide) (m/z: 448.1256, RT: 17.64 min, 0.22-fold). These metabolites all have p-values of <0.05. B) Plots of individual metabolites perturbed after AN5568 treatment. There was an enrichment in metabolites involved in L-methionine metabolism. WT; wild type untreated, AN5568; wildtype treated for six hours at 10x EC_50_. C) A schematic to show the metabolites involved in methyltransferase reactions. Red ‘x’ indicates the typical S-adenosyl-L-methionine-dependent methyltransferase reaction potentially affected by the benzoxaborole.

Metabolomics analysis, using LC-MS, was carried out on *T*. *b*. *brucei* treated with AN5568 for six hours at a concentration of 1.9 μM (10-fold EC_50_). This time point was chosen as metabolic alterations occur much quicker than those of the genome or transcriptome. In addition, toxic compounds can lead to widespread metabolic perturbation, which would mask the effects of specific target inhibition as explained above. In this experiment, a total of 840 peaks were tentatively identified and considered as metabolites (Fig 1). Of these, 50 were significantly altered after drug treatment (Log_2_ fold-change = <-1, >1, P<0.05 [*t*-test]). These included a range of metabolites involved in carbohydrate metabolism and lipid metabolism, but mostly amino acid metabolism (S1 Table).

Following treatment, we observed an enrichment in metabolites involved in methionine metabolism (Fig 1), suggesting methyltransferases (MTase) as potential targets. The most significant of these changes were in the levels of S-adenosyl-L-methionine (AdoMet) (*m/z*: 398.1374, retention time (RT): 18.01 min, 7.67-fold, *P* = 0.0096) and 5’-methylthioadenosine (5’-MTA) (*m/z*: 297.0838, RT: 7.75 min, 6.17-fold, *P* = 4.61×10^−6^) (Fig 1). The levels of adenine, a purine nucleobase that is also formed as a byproduct of AdoMet degradation, were also increased (*m/z*: 135.0546, RT: 7.71 min, 12.55-fold, *P* = 0.0045) (Fig 1). The intermediate decarboxylated AdoMet (dcAdoMet), a key aminopropyl donor used in polyamine biosynthesis was not detected by LC-MS.

These metabolites are all associated with the degradation of methionine, in addition to the recycling of this amino acid, which occurs in a cyclic metabolic pathway known as the Yang cycle, methionine salvage, or the 5’-methylthioadenosine (MTA) cycle [29, 30]. This pathway is crucial in maintaining a source of methyl groups for methyltransferase reactions as well as other processes such as polyamine biosynthesis [31]. It is currently unknown whether the complete Yang cycle is functional in *T*. *brucei*, although it is thought the parasite relies on uptake of exogenous L-methionine, rather than recycling the amino acid [32]. Nevertheless, metabolomics data were searched for further metabolites involved in the MTA cycle.

Interestingly, one peak was putatively identified as 1,2-dihydro-5-(methylthio)pent-1-en-3-one (Fig 1A, red metabolite #2), which was significantly increased in AN5568-treated cells. This metabolite plays a role in the regeneration of L-methionine through 2-oxo-4-methylthiobutanoate (KMTB) [33]. The metabolomics data were further mined for 2-oxo-4-methylthiobutanoate, and a mass consistent with this metabolite appeared to decrease post-treatment (S1 Table).

Whilst AdoMet was increased after AN5568 treatment, there was no corresponding decrease in levels of S-adenosyl-L-homocysteine (AdoHcy), the by-product of methylation reactions (Fig 1). Whilst there was no explanation for this observation, it is likely that homeostatic processes are at play. Indeed, the activity of S-adenosyl-L-homocysteine hydrolase (SAHH), the enzyme that catalyses the breakdown of AdoHcy into L-homocysteine and adenosine, is strictly regulated in other organisms [34]. L-homocysteine similarly remained unchanged in the treated sample group. Importantly, two peaks corresponding to AN5568 were found, one for the parent ion and one for the boron-10 isotope (S1 Table).

Most other changes observed after AN5568-treatment were in metabolites involved in amino acid metabolism (S1 Table), including increases in keto-arginine, modified lysines (mono-, di- and tri-methyl-L-lysine and acetyl-L-lysine) and aminoacetone as well as decreases in L-aspartate, L-proline, D-glucosamine 6-phosphate, L-carnitine and O-acetyl-L-carnitine (S1 Table).

### AN5568 provokes similar changes in procyclic *T*. *b*. *brucei* metabolism

**Fig 2:**
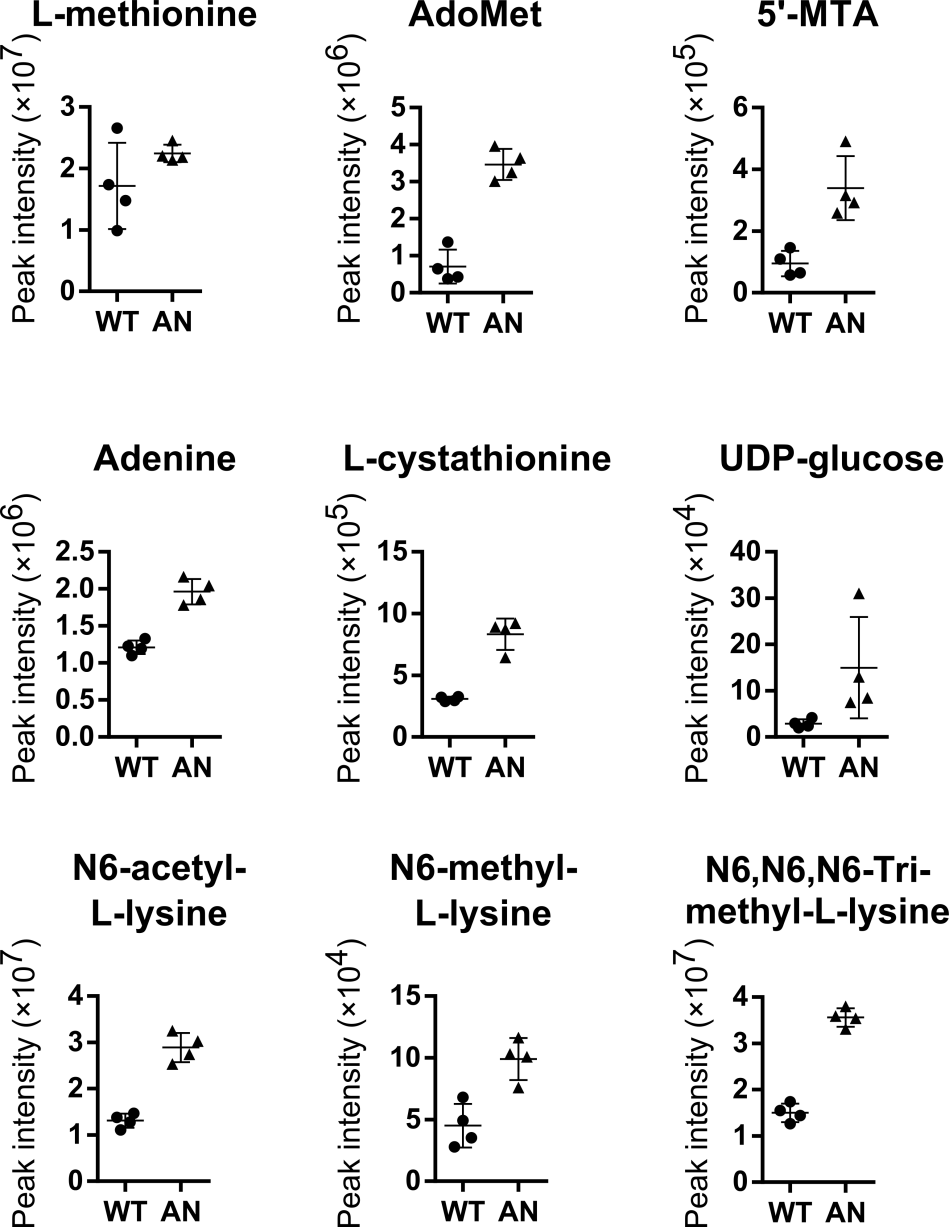
Metabolic changes in PCF *T*. *b*. *brucei* after AN5568 treatment. Plots of individual metabolites perturbed after AN5568 treatment. WT; wild-type untreated, AN5568; wildtype treated for eight hours at 15 μM (10x EC_50_).

PCF Lister 427 cells were exposed to 15 μM (10x EC_50_) AN5568 to investigate whether benzoxaborole treatment induced similar changes in the metabolism of PCF cells to those observed in BSF cells (Fig 2). Important to note was the higher concentration of AN5568 in PCF cultures and a longer duration of drug exposure of 8 hours. The metabolic changes in PCF cells were found to be similar to those in BSF cells, indicating that the molecular mechanisms of drug action might also occur in the PCF stage.

Once again, the most significant changes involved L-methionine and AdoMet metabolism (Fig 2). AdoMet (*m/z*: 398.1386, RT: 13.88 min) and 5’-MTA (*m/z*: 297.0898, RT: 7.69 min) were both highly increased after drug treatment. In addition, mono-, di- and tri-methylated lysines were again observed at high abundance. Finally, PCF cells also underwent changes in glycoprotein metabolism, as indicated by increased levels of UDP-glucose (Fig 2).

### Comparative analysis of AN5568 activity to Sinefungin, a non-specific methyltransferase inhibitor

To investigate the hypothesis that MTases are targeted by AN5568, we compared the metabolomes of *T*. *b*. *brucei* cells treated with the non-specific AdoMet-dependent MTase inhibitor sinefungin, to those of benzoxaborole-treated cells. Sinefungin is an AdoMet analogue containing an aminomethylene group in place of the methylated sulfonium group that typically acts as the methyl donor. It competes with AdoMet for MTase-binding and thereby inhibits AdoMet-dependent MTases in a non-selective manner [35, 36].

To determine whether AN5568 and sinefungin target similar pathways *in vitro*, isobologram experiments were conducted. Firstly, EC_50_ concentrations were calculated to be 193 nM and 1 nM for AN5568 and sinefungin respectively. Subsequently, a fixed ratio isobologram, as outlined previously by Fivelman and colleagues [37], was carried out to observe whether the effect of using the two compounds simultaneously was greater (synergism) or less than (antagonism) the sum of the two compounds used separately. For each ratio (outlined in the methods), the fractional inhibitory concentration (FIC) was calculated by dividing the EC_50_ of one drug in combination with the other, by the EC_50_ of that drug used alone. The sum of FIC was calculated by adding the FIC for one drug to that of the other for each ratio. Synergism can be defined as a mean FIC <0.5, whilst antagonism is likely occurring when FIC>1. Anything in between suggests the compounds do not interact.

**Fig 3:**
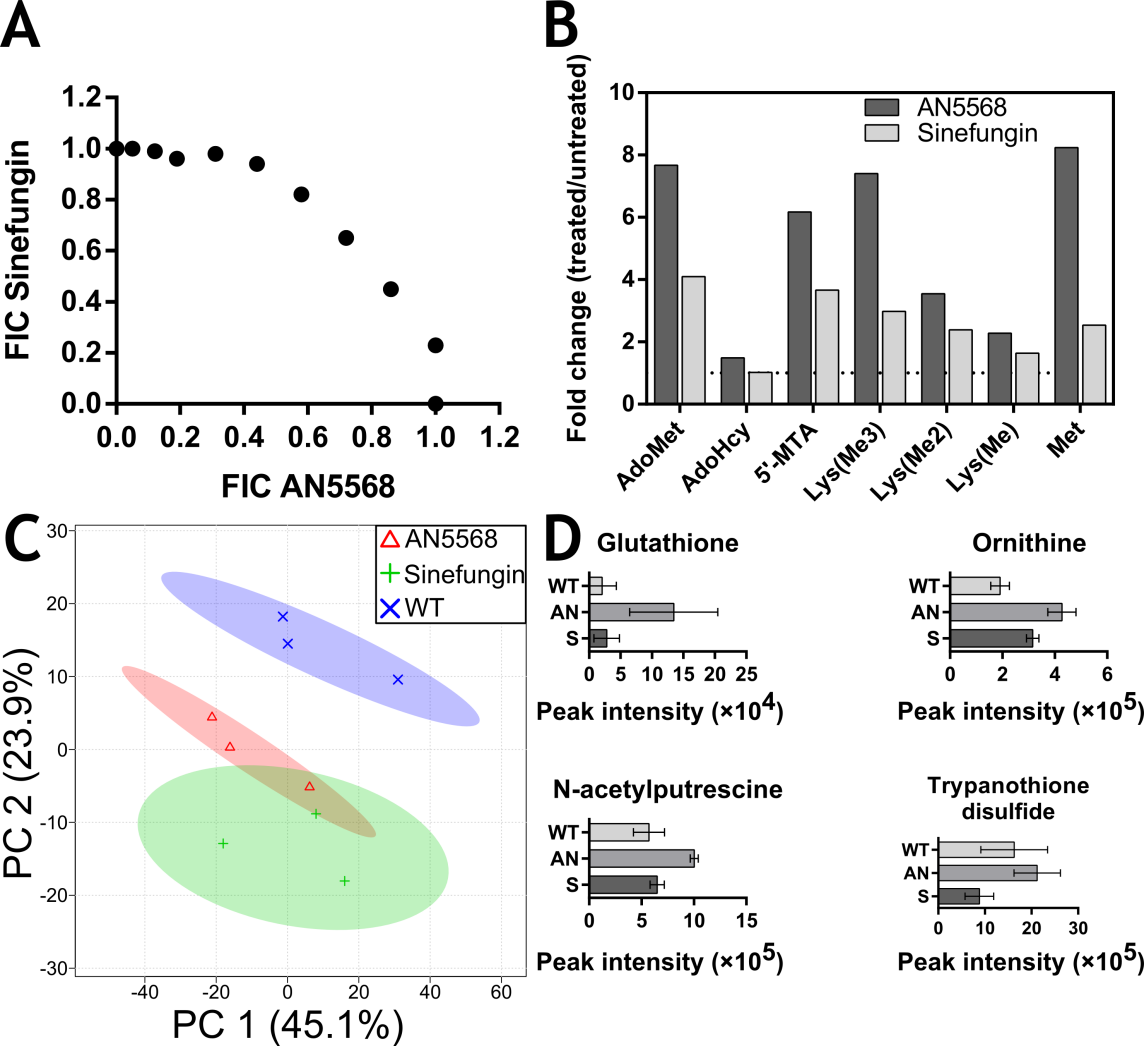
Comparative analyses of *T*. *b*. *brucei* treated with AN5568 and sinefungin, a non-specific methyltransferase inhibitor. A) A fixed-ratio isobologram analysis was conducted using sinefungin and AN5568. B) Comparative metabolomics analysis was carried out to assess any common metabolic alterations between sinefungin- and AN5568-treated cells. A principal component analysis (PCA) plot of the 3 sample group was generated. C) Individual metabolites were compared in terms of their fold change over a control group. D) AN5568 treatment leads to increased abundance in components of the trypanothione biosynthesis pathway, whilst sinefungin treatment does not.

A scatter plot of FIC values showed higher FICs when the compounds were used in combination (Fig 3A). The mean total fractional inhibitory concentration (ΣFIC) for AN5568 and sinefungin was calculated to be 1.21, suggesting slight antagonism between the two compounds.

Metabolomics experiments were subsequently carried out, and a principal component analysis (PCA) plot of three sample groups, wild-type, AN5568-treated and sinefungin-treated, revealed an association between the two drug-treated sample groups, which separated from the wild-type group (Fig 3B). Whilst the wild-type samples separated, both drug-treated samples overlapped, thereby suggesting similarities between the two sample groups. Indeed, the main metabolic perturbations observed in AN5568-treated cells were also apparent in cells treated with sinefungin (Fig 3C, S1 Table). Most notably, increases in AdoMet and 5’-MTA were reproducible in both treatment groups. In addition, sinefungin-treated cells also exhibited increased abundance of mono-, di- and tri-methylated lysines, as well as adenine, all of which mirrored the metabolic changes in AN5568-treated cells.

Interestingly, AdoHcy remained unchanged after sinefungin treatment (Fig 3C), suggesting that methyltransferase inhibition does not lead to decreased levels of AdoHcy that might be expected from inhibition of this class of enzymes. This might be due to downstream metabolite regulation of AdoHcy-hydrolase as previously mentioned [34].

There were also marked contrasts between AN5568- and sinefungin-treated cells. For example, AN5568-treated cells clearly exhibited changes in the trypanothione biosynthesis pathway, whilst these changes did not occur after sinefungin treatment (Fig 3D).

### Distribution of carbon originating from L-methionine in the *T*. *b*. *brucei* metabolome is not altered after AN5568 treatment

To investigate whether any alterations in methylation patterns could be observed across the metabolic network, we carried out further LC-MS utilising Creek’s Minimal Medium (CMM) [38]. Adding 100% [U-^13^C]-L-methionine to CMM allowed the observation of the dissemination of the isotope-labelled carbon atoms to be observed. As previously, cells were treated with 10× EC_50_ AN5568, and incubated for 6 hours under normal *in vitro* conditions.

**Fig 4:**
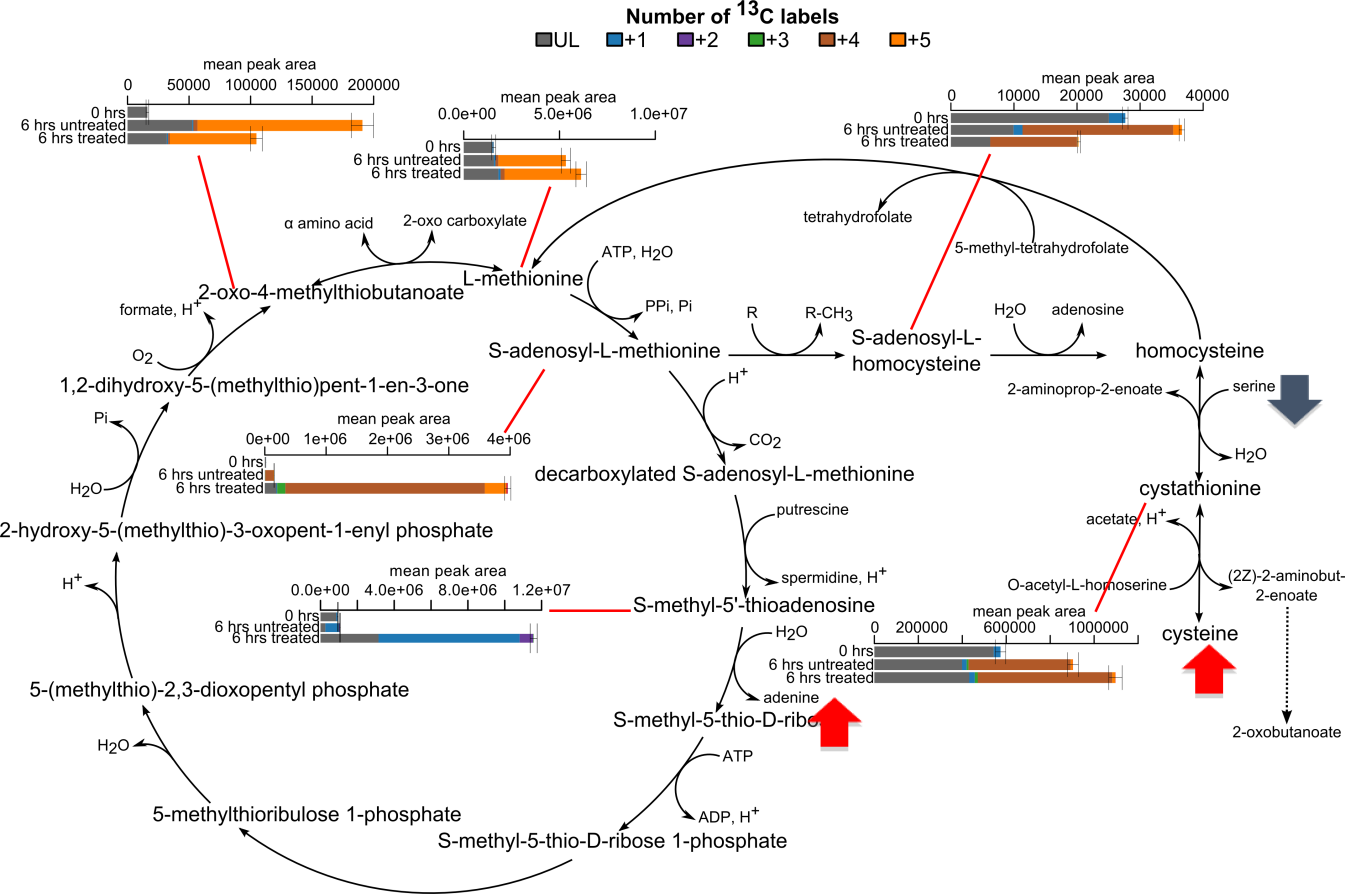
Tracing ^13^C distribution in AN5568-treated cells incubated with ^13^C-U-L-methionine. Cells were incubated with 200 μM [U-^13^C]-L-methionine for 6 hours. In addition, one sample group was incubated with 1.9 μM (10× EC_50_) AN5568. Metabolites involved in L-methionine metabolism detected in this experiment were overlaid onto metabolic maps. In cases where ^13^C isotopes were detected, graphs incorporating the percentage labelling are shown. The methionine salvage pathway (Yang cycle) is shown.

A total of 25 peaks containing ^13^C isotopes were detected (17 in negative mode, 8 in positive mode). Whilst the previously observed changes in methionine metabolism were reproducible, no changes in carbon distribution were observed between AN5568-treated and untreated cells (Fig 4).

As expected, metabolic changes were consistent with previous experiments but intracellular L-methionine was not 100% labelled, suggesting that uptake is relatively slow. Hasne and colleagues previously showed L-methionine uptake to be transporter mediated, and in BSF *T*. *b*. *brucei* the transporter exhibits a V_max_ of 28.8 ± 0.1 nmol/min/10^8^ cells [32].

Interestingly, whilst only small amounts of AdoMet and 5’-MTA were detected in untreated samples, they showed almost 100% labelling (Fig 4A), suggesting these metabolites all originate from L-methionine. In contrast, these metabolites were only ~75% labelled in drug-treated samples, suggesting they might be recycled, or the build-up initiates very quickly after drug treatment. AdoHcy was also detected, with four ^13^C labels, as predicted. In addition, L-cystathionine possessed four ^13^C labels, and was found to increase, as seen previously (Fig 4A).

Whilst arginine methylation was not observed in wild-type or drug-treated samples, methylated lysines that were seen to increase in previous experiments did show stable isotope labels (S1 Fig), demonstrating that the carbon atoms in the methyl groups originated from L-methionine. Lysine methylation has been shown to be involved in the degradation pathway of this amino acid, which is ultimately converted to L-carnitine [39]. However, in neither sample group was L-carnitine found to exhibit ^13^C labelling (S1 Fig), indicating a separate source of this metabolite in *T*. *b*. *brucei*. Indeed, it was previously shown that the parasite has a high rate of L-carnitine uptake from serum [40].

### AN5568 treatment affects glycoprotein metabolism, but does not lead to impaired VSG synthesis

**Fig 5:**
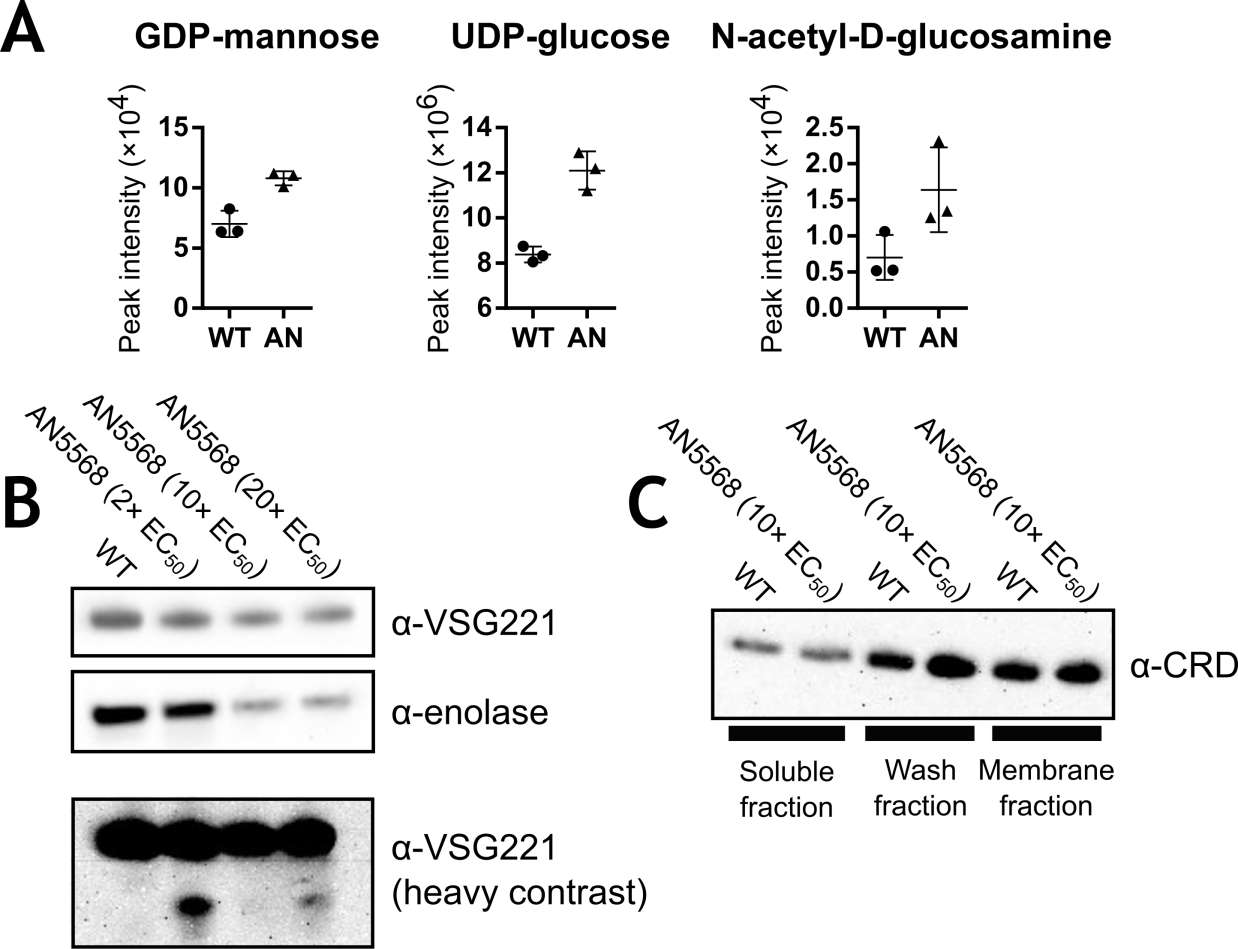
VSG biosynthesis in AN5568-treated cells. A) Three metabolites, all known to be involved in glycoprotein biosynthesis, were found to be at higher levels in AN5568-treated cells. B) Western blots carried out on protein lysates taken from AN5568-treated cells at a 6-hour time-point showed no significant changes in VSG expression at this time-point. When the contrast of the resulting blot was increased, an extra band was seen in the 2× EC_50_-treated sample. C) In addition to VSG expression, blots were probed with a VSG cross-reacting determinant (VSG XR) antibody. For this experiment, protein lysates were fractionated to isolate the membrane fraction and separate it from the soluble fraction. Most of the cellular GPI is found in the membrane, and no changes in expression could be seen in cells treated with 10× EC_50_ for 6 hours. WT; untreated wild-type cells, AN5568; wild-type cells treated with AN5568 for 6 hours at 10× EC_50_.

Several metabolites putatively identified as components of glycoprotein metabolism were elevated in benzoxaborole-treated cells. These included GDP-mannose, N-acetyl-D-glucosamine and UDP-glucose (or UDP-galactose) (Fig 5A). These metabolites are important components in the production of glycosylphosphatidylinositol (GPI) anchors that attach the variant surface glycoproteins (VSG) to the trypanosome cell surface. To determine whether VSG biosynthesis was affected by AN5568 treatment, Western blots were generated using the Lister 427 line, which expresses VSG221 [41]. Firstly, lysates from cells treated with 10× EC_50_ AN5568 for 6 hours were probed with an αVSG221 antibody, and subsequently an α-enolase control (Fig 5B). No change in VSG221 expression was observed, although there appeared to be reduced expression of the endogenous control. Indeed, further analysis of the metabolomics data showed decreased levels of both the substrate and product (2-phospho-D-glycerate and phospho*enol*pyruvate respectively) of this enzyme, for reasons we could not ascertain (S1 Table).

A further blot was generated using VSG cross-reacting determinant antibodies (VSG XR) (Fig 5C) [42]. For this experiment, cell lysates were separated into membrane and soluble fractions. Again, no changes were observed between the control and drug-treated samples, suggesting VSG biosynthesis is not impaired on a protein level.

### Comparing metabolic phenotypes of AN5568 treatment and ER stress

**Fig 6:**
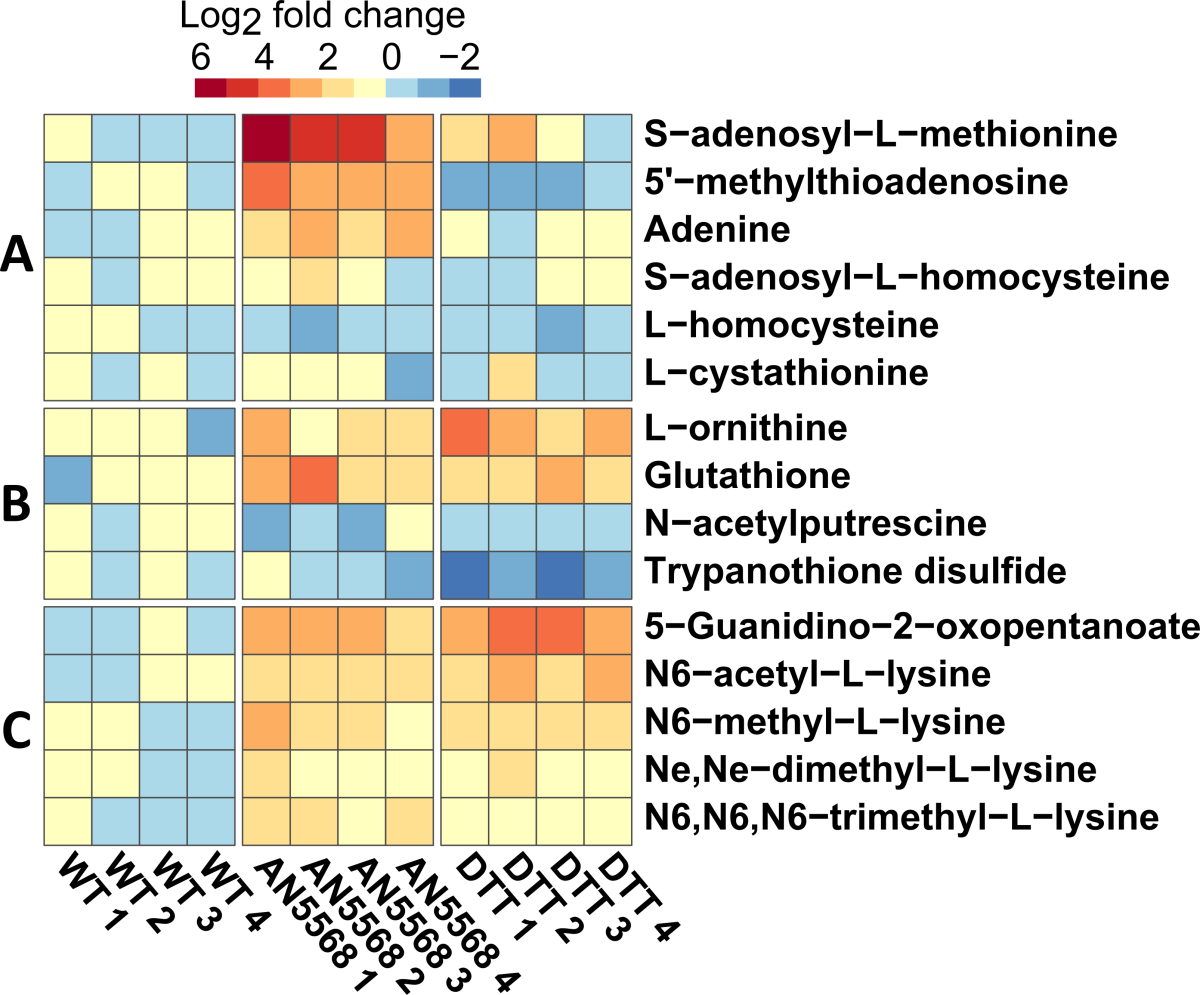
Comparing metabolic profiles of DTT and AN5568 treated cells. A heatmap was generated to highlight similarities and differences in metabolism after treatment with the ER stress inducer dithiothreitol (DTT) and AN5568. A) AdoMet metabolism was unaffected by DTT-treatment. B) Components of the oxidative stress response pathway were altered after both treatments. In particular, DTT treatment led to a significant decrease in trypanothione disulphide. C) Both DTT and AN5568 treatment result in increased levels of modified lysines and arginines, suggesting these are part of a conserved trypanosomatid stress response. WT; wild-type untreated, AN5568; wild-type cells treated with AN5568 for 6 hours at 10× EC_50_, DTT; wild-type cells treated with DTT for 6 hours at 10× EC_50_.

A major target for AdoMet donated methyl groups in *T*. *b*. *brucei* is the spliced leader cap RNA structure that is spliced to all mature messenger RNAs in these cells. Recent publications have highlighted the presence of a unique ER stress response pathway in trypanosomatids [43–45]. In response to unfolding proteins, and to ER stress inducers such as dithiothreitol (DTT), a programmed cell death known as the spliced leader silencing (SLS) pathway is activated, leading to programmed inhibition of spliced leader *trans*-splicing, as well as a reduction in total RNA, increased cytoplasmic Ca^2+^ and DNA fragmentation [43]. Presumably, this would also result in significant loss of RNA methylation. We therefore sought to compare the metabolomic profiles of DTT and AN5568 treated cells in an LC-MS experiment, in order to determine whether the AdoMet signature was a specific response to the benzoxaborole treatment, or a non-specific stress response (Fig 6).

No changes were observed in AdoMet (*m/z*: 398.1381, RT: 13.72 min, 2.53-fold, *P* = 0.230) and adenine (*m/z*: 135.0545, RT: 9.48 min, 1.37-fold, *P* = 0.190), after DTT-treatment (Fig 6A). Furthermore, 5’-MTA (*m/z*: 297.0889, RT: 6.70 min, 0.54-fold, *P* = 0.034) was decreased, compared to wild-type control, not increased as observed in AN5568 treatment.

DTT treatment also led to metabolic changes in stress responses such as the trypanothione biosynthesis pathway (Fig 6B). Modified lysines were increased, in a similar fashion to AN5568 treatment. In particular, there was increased abundance of N6-acetyl-L-lysine (*m/z*: 188.1162, RT: 11.62 min, 4.26-fold, *P* = 0.0021), N6-methyl-L-lysine (*m/z*: 160.1211, RT: 18.99 min, 2.80-fold, *P* = 0.0004) and N6,N6,N6-trimethyl-L-lysine (*m/z*: 188.1528, RT: 18.09 min, 1.62-fold, *P* = 0.013) (Fig 6C), suggesting these changes are not unique to AN5568 treatment, but potential indicators of metabolic responses to stress. Further similarities were also found in keto-arginine (S2 Table).

### *In silico* analysis of the *T*. *brucei* methyltransferome

Bioinformatics analysis of the entire kinase complement in the Tri-Tryps (*T*. *brucei*, *T*. *cruzi* and *Leishmania spp*.) has greatly aided the identification of novel therapeutic targets, and driven research into this class of enzymes [46]. Methyltransferases (MTases) are a different class of highly specialised enzymes involved in the methylation of a large variety of substrates, which carry equal potential as therapeutic targets. However, the *T*. *brucei* methyltransferase complement has not been studied in great detail, with only ~25 experimentally characterised. Indeed, only two large-scale MTase studies have previously been reported [47, 48]. Given the metabolomics data generated, we sought to apply this *in silico* approach to characterise the *T*. *brucei* “methyltransferome” (MTome), with the aim of identifying potential AN5568 targets.

**Fig 7:**
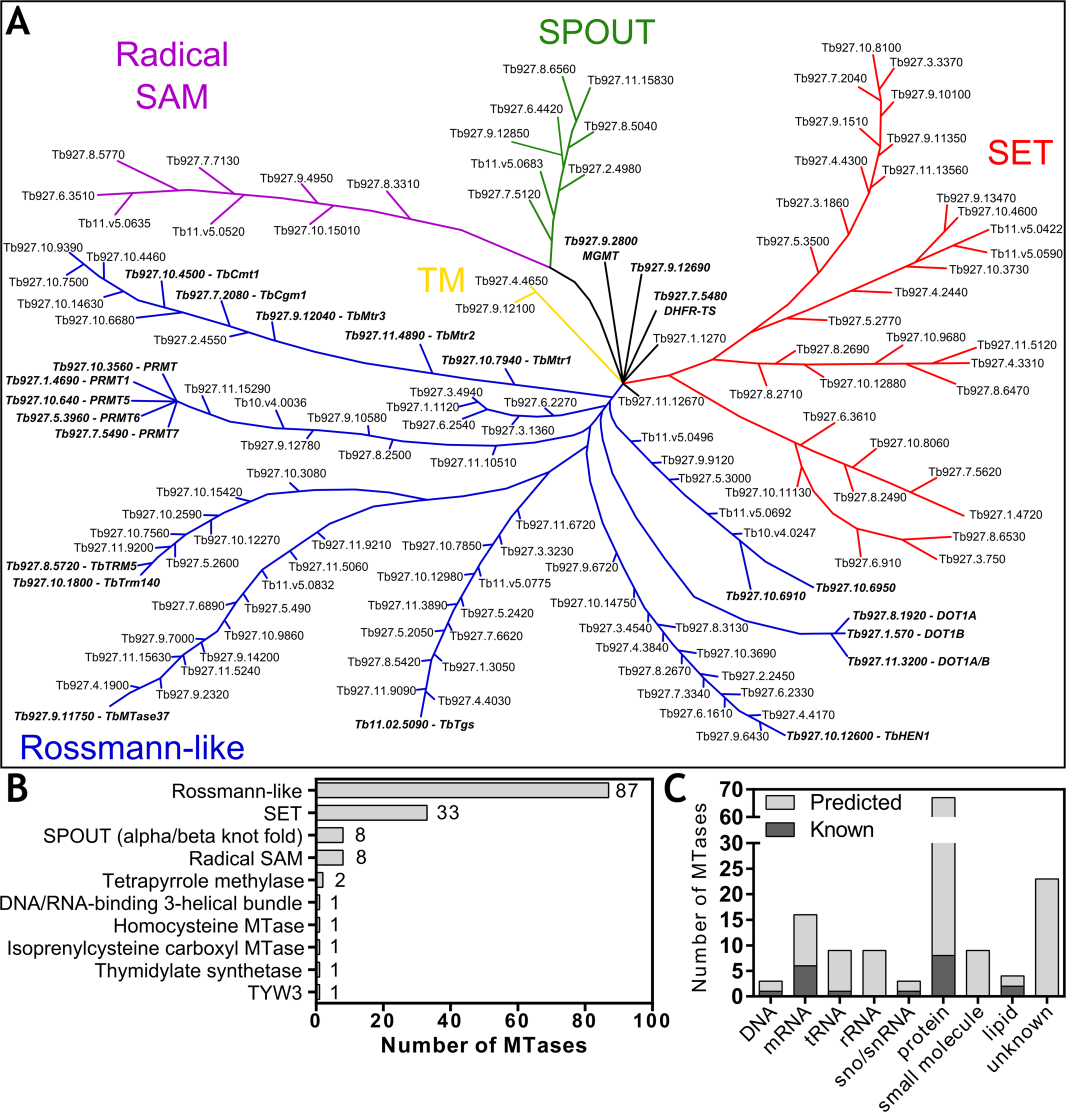
The *T*. *brucei* “methyltransferome”. A) A stylised tree depicting the entire methyltransferase complement of *T*. *brucei*, clustered based on domain class as designated by Schubert and colleagues [52]. The arrangements of genes and branch lengths carry no significance as methyltransferases are highly diverse, making generation of a phylogenetic tree challenging. Gene IDs in bold italics have been previously characterised. Abbreviations: TM: Tetrapyrrole methylase. B) Number of members in each class of methyltransferase. Enzymes containing Rossmann-like folds and SET domains are numerous compared to the remaining classes. C) Methyltransferases grouped by the substrate they methylate. The majority of MTases studied include spliced leader RNA MTases and protein arginine MTases. SET domain-containing proteins are likely lysine MTases.

Using a combination of interpro and pfam [49–51], a total of 143 genes containing MTase domains were identified (Fig 7), the majority of which contained additional conserved domains involved in substrate binding, transmembrane domains and localisation signalling domains (S3 Table). This list was categorised based on major folds present in the MTase domains, similar to the methodology used in the Wlodarski and Petrossian & Clarke studies [47, 48].

The majority of MTases (87/143) were found to exhibit Rossman-like folds. Most of the experimentally characterised methyltransferases are present in this group, including arginine methyltransferases, DOT methyltransferases and proteins involved in spliced leader methylation (Fig 7A). Furthermore, this group contains lipid MTases as well as rRNA and tRNA MTases, most of which remain uncharacterised.

Another group with many representatives are proteins containing the conserved SET domain (Fig 7A & 7B). These 33 genes are likely to be involved in protein lysine methylation, in particular methylation of histones [53]. This group is far larger than expected (The *S*. *cerevisiae* genome encodes 12 SET domain MTases [47]), and could be indicative of the parasite’s need to alter global gene expression throughout the varying life cycle stages. Further work is required to confirm whether these proteins are true MTases. Indeed, none of the *T*. *b*. *brucei* SET domain MTases have been experimentally characterised, yet proteins of this class have been identified as therapeutic targets in cancer, and other protozoan parasites such as *Plasmodium* [54], and could present an interesting group of trypanocidal candidate targets.

Like the *S*. *cerevisiae* MTome, the *T*. *brucei* genome also contains genes for other MTase family members, including SPOUT domain MTases (8 genes), tetrapyrrole methylases (2 genes), a DNA/RNA-binding 3-helical bundle MTase (1 gene) a thymidylate synthase (1 gene) a homocysteine MTases (1 gene), isoprenylcysteine carboxyl MTase (1 gene) and a TYW3 MTase (1 gene) (Fig 7). Finally, whilst not genuine methyltransferases, we chose to include the 8 predicted radical SAM enzyme family. These proteins typically cleave AdoMet to generate a 5’-deoxyadenosyl 5’-radical [55].

We attempted to overexpress several of the genes identified in the MTome screen based on their essentiality in an RNAi screen [56] (Gene IDs: Tb927.10.3080, Tb927.10.7560, Tb927.10/7850, Tb927.5.2050, Tb927.8.5040 and Tb927.6.2270). Whilst overexpression of some MTases was achievable, their overexpression did not alter parasite sensitivity to AN5568, although in the case of Tb927.8.5040 and Tb927.10.7850, resistance against sinefungin was noted (S2 Fig).

### Microscopy analysis of AN5568 treated BSF cells

**Fig 8:**
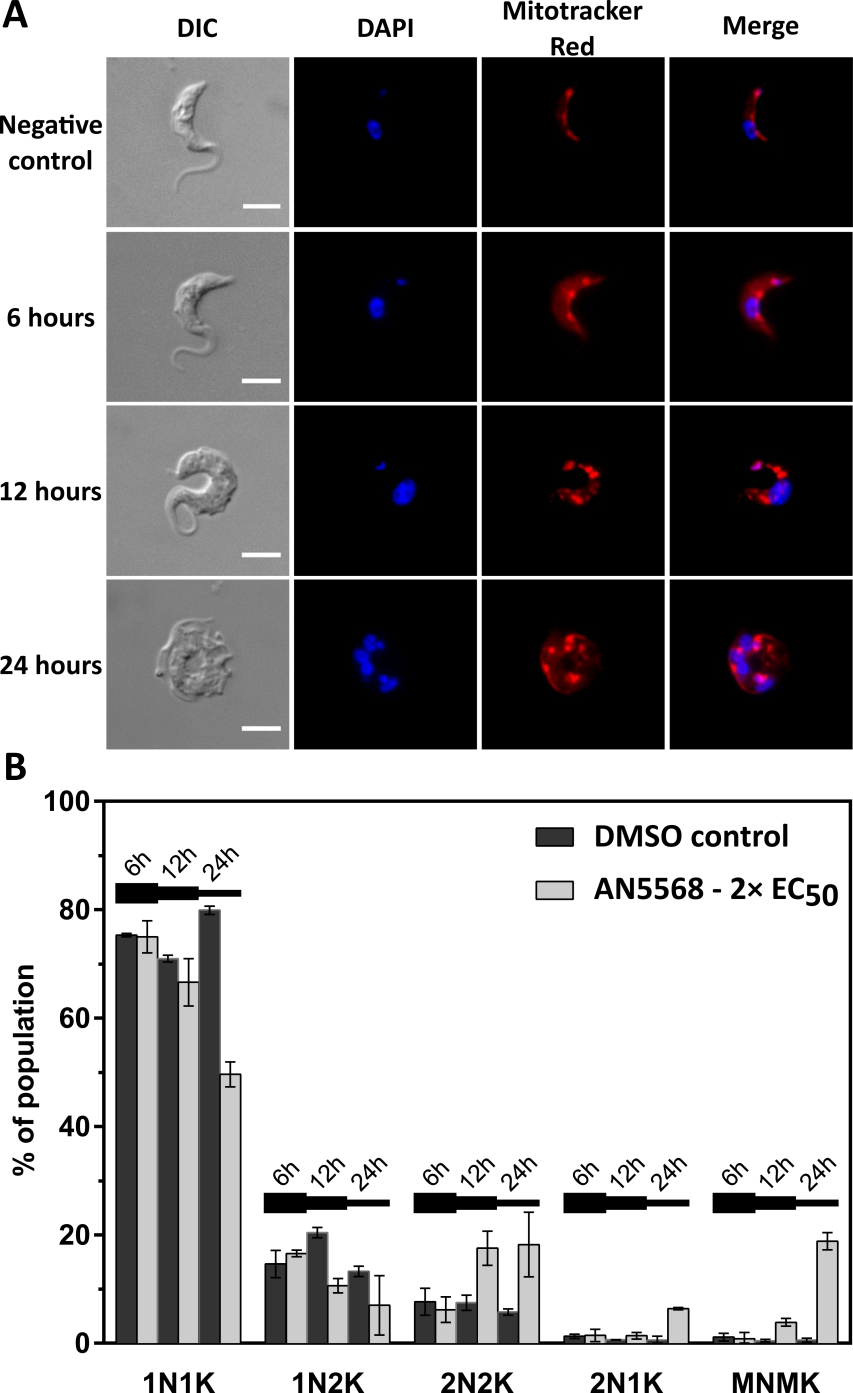
Effect of AN5568 treatment on *T*. *b*. *brucei* cell division and morphology. *T*. *b*. *brucei* cells were incubated with 2× EC_50_ concentration of AN5568 and cells were isolated for analysis by microscopy at specific time points. A) After 6 hours of drug exposure, cells appeared normal under both direct light and DAPI, whilst mitochondria were swollen. This phenotype was exaggerated by 12-hours post-treatment. After 24 hours, DAPI staining indicated a build-up of DNA-containing compartments in a significant number of cells, suggesting a cytokinetic defect. Scale bar represents 5 μm. B) Cell cycle analysis was carried out by counting the numbers of nuclei and kinetoplasts in AN5568-treated cells. Drug-treated cells were compared to a DMSO control at 6, 12 and 24 hour time-points, as indicated by the horizontal lines.

Morphological changes occurring during AN5568 treatment of *T*. *b*. *brucei* were investigated using fluorescence microscopy. The nuclear and kinetoplastid DNA were stained using DAPI, whilst Mitotracker was used to visualise the mitochondrion (Fig 8). Time-course experiments were carried out on BSF *T*. *b*. *brucei* using a single dose of AN5568 at 2× EC_50_ (380 nM). Wild-type controls were supplemented with an equal volume of DMSO.

No significant changes in morphology were observed 6 hours post-treatment (Fig 8A). However, by 12 hours, most cells exhibited a rounded shape indicating adverse responses to the compound (Fig 8A). In addition, DAPI staining revealed several nucleolar spots visible after 12 and 24 hours, which could indicate a mitotic block. Interestingly, Mitotracker staining showed that rather than a long, single, elongated mitochondrion typical of BSF *T*. *brucei*, the organelle appeared patchy and swollen (Fig 8A). After 24 hours of 2× EC_50_ treatment, the aforementioned phenotypes were far more pronounced and cells were rounded, with multiple flagella and lack of flagellar extension. Furthermore, DAPI highlighted the presence of multiple DNA-compartments.

To test whether AN5568 treatment affected *T*. *b*. *brucei* cell cycle and/or nuclear and kinetoplast replication, NK analysis was conducted (Fig 8B). Percentages were calculated for cells containing 1 of each organelle (1N1K); 1 nucleus and 2 kinetoplasts (1N2K); two of each organelle (2N2K); 2 nuclei and 1 kinetoplast (2N1K), which suggests a defect in kinetoplast replication; and finally, cells containing more than 2 of each organelle (MNMK).

The NK results mirrored the morphology analyses, and parasites exhibited no significant changes in nuclear and kinetoplast number after 6 hours. However, by 12 hours, almost 20% of cells contained 2 kinetoplasts and 2 nuclei, almost double the percentage of untreated parasites (Fig 8B).

## Discussion

The benzoxaborole AN5568 (SCYX-7158) is a promising lead compound for treatment of HAT, an infectious disease caused by *T*. *brucei* subspecies. Whilst the drug is currently awaiting phase III clinical trials, the MoA is as-of-yet unknown. We sought to use a metabolomics-based approach to reveal the metabolic perturbations induced by AN5568 in the lab-adapted Lister 427 strain of *T*. *b*. *brucei*.

AN5568 treatment induced 50 significant metabolic perturbations in BSF trypanosomes, with most identified as components of amino acid metabolism. In particular, there was an enrichment of metabolites involved in L-methionine and AdoMet metabolism, leading us to hypothesize that an MTase could be targeted. Indeed, AN5568 exhibits antagonism with sinefungin, a competitive AdoMet-dependent MTase inhibitor.

Further mass spectrometry analyses were undertaken to compare metabolic profiles of sinefungin, a known methyltransferase inhibitor and AN5568-treated parasites. These experiments identified AdoMet and 5’-MTA as key players in methyltransferase inhibition.

Stable isotope-labelled [U-^13^C]-L-methionine was introduced to *in vitro* culture, in an attempt to resolve the aforementioned metabolic changes. However, to our surprise, fewer than 20 metabolites were found to incorporate carbon from L-methionine. This suggests that a) L-methionine is not an essential source of carbon in terms of small molecule metabolism, and b) carbon in methylation reactions is mainly transferred to larger molecules that could not be detected by LC-MS, probably proteins. Furthermore, the absence of labelled methyl-arginine in either untreated controls or drug-treated cells was surprising, and suggests that arginine methylation is either a slower process than, for example, lysine methylation, or the methyl groups used to methylate arginine do not originate from L-methionine or indeed AdoMet. In addition, these processes could occur in different subcellular localisations or compartments.

Interestingly, the metabolic profile of DTT treated cells bore some similarities to those treated with AN5568, suggesting that *T*. *b*. *brucei* activates the conserved SLS pathway in response to the benzoxaboroles, although DTT may also impair cellular redox balance and induce other stresses in common with AN5568. These experiments were performed in order to see whether the loss of trans-splicing associated with the SLS pathway might lead to accumulation of AdoMet and related metabolites too, although it did not.

Several findings presented here are in agreement with a previous study of AN5568 MoA in *T*. *b*. *brucei* [25]. Most notably, inhibition of cytokinesis was observed in both studies [25] (Fig 8). However, many drugs affect the ability of *T*. *b*. *brucei* to divide, and it is therefore not indicative of a particular mode of action for this compound. Interestingly, in the context of these data, Jones and colleagues found genomic deletions in one resistant cell line in a region corresponding to the AdoMet decarboxylase (AdoMetDC) array. However, if AdoMetDC was the benzoxaborole target, treatment would hypothetically lead to reduced levels of 5’-MTA which was not seen (Fig 1).

Based on the data presented here, it seems that AN5568 has a significant effect on methionine metabolism, and induces a stress response similar to the spliced leader silencing pathway. However, we have not been able to assign these effects directly to a specific enzyme or particular methyltransferase reaction.

This study also presents, to our knowledge, the first collection of all predicted MTases in the *T*. *brucei* genome. Using previous studies of *H*. *sapiens* [48] and *S*. *cerevisiae* [47] as a guide, as well as publicly available pfam annotations from TriTrypDB [57], a dataset was generated based on MTase class and fold. MTases are highly diverse and uniquely specific depending on their biological function, and many are essential in protein synthesis, regulation of gene expression, spliced leader methylation, and regulation of the cell cycle. They therefore present a protein family of therapeutic interest.

One interesting finding in this regard is the large number of SET domain MTases which are predicted lysine methylators, predicted in the *T*. *brucei* genome. It is likely that many of these enzymes play crucial roles in histone modulation and maintenance of gene expression throughout the extremely diverse life cycle stages of the parasite. The dataset requires further refinement and expansion to include *T*. *cruzi* and *Leishmania spp*. Furthermore, the majority of these proteins remain uncharacterised experimentally. We hope this dataset will inspire further work on a group of proteins that in other settings, such as cancer biology, has recently generated intense interest.

Although we now reveal metabolomics changes to trypanosomes treated with AN5568 we cannot attribute a mode of action to the drug from this data, in common with the other high throughput analysis on proteins that bind the drug and genetic differences in parasites selected for resistance [18]. However, the results outlined here suggest that AN5568 action is not in a catabolic or anabolic pathway, similar to other members of the benzoxaborole family [15, 16, 19, 21]. Further studies, for example, seeking genome scale over-expression libraries will be required to definitively find the cellular target of the drug.

## Methods

### *T*. *b*. *brucei in vitro* culture

For BSF culture, the Lister 427 strain was used and maintained at 37°C, 5% CO_2_. Cells were cultured in HMI-9, supplemented with 10% foetal bovine serum (FBS). For [U-^13^C]-L-methionine experiments, Creek’s Minimal Media was used [38], and isotope labelled metabolites were added upon initiation of time-course experiments. Procyclic form culture also utilised Lister 427 (also identified as 29–13). PCFs were cultured in SDM-79 (Gibco, Cat#: 07490916N) [58], supplemented with 10% FBS and 7.5 μg/mL hemin. PCF cell density was maintained between 5 × 10^5^ cells/mL and 1 × 10^7^ cells/mL and grown at 27°C.

For overexpression of methyltransferases, BSF T. brucei Lister 427 were transfected with methyltransferase open reading frames inserted into the pURAN vector. Transfection was carried out as previously described [56]. Drug sensitivity in successfully transfected cell line was tested by alamar blue.

### Further parasite *in vitro* culture

*T*. *congolense* bloodstream form, strain IL3000, were cultured in TcBSF3 a recently developed culture medium. Cultures were supplemented with 20% goat serum (Gibco) and 5% serum plus (Sigma). Density was maintained between 5 × 10^4^ and 2 × 10^6^ cells/mL and cultured were incubated at 34°C, 5% CO_2_.

*Leishmania mexicana* promastigotes were cultured in a modified Minimum Eagle’s medium, termed HOMEM, supplemented with 10% FBS and 1% penicillin/streptomycin [59]. Cultures were maintained at 25°C.

### Alamar blue assays

To obtain *in vitro* EC_50_ values for specific compounds targeting *T*. *b*. *brucei*, the alamar blue assay was applied in 96-well plates (adapted from [60]). Compounds were added starting with the highest concentration (typically 100 μM) and serially diluted 1:2 over either 23 wells, leaving one negative control. Subsequently, BSF *T*. *b*. *brucei* cells were added at a final density of 2 × 10^4^ cells/mL. Plates were incubated at 37°C, 5% CO_2_ and 20 μL of alamar blue reagent (resazurin sodium salt, 0.49 mM in 1× PBS, pH 7.4) was added to each well after 48 hours. Reduction of the alamar blue reagent was measured as a function of cell viability on a BMG FLUOstar OPTIMA microplate reader (BMG Labtech GmbH, Germany) with λ_excitation_ = 544 nm and λ_emission_ = 590 nm. The raw values were plotted against the log value of each concentration of drug or compound, and EC_50_ values were calculated using a non-linear sigmoidal dose-response curve. Each assay was performed in duplicate, and the final EC_50_ values presented represent a mean of three or four independent experiments.

For PCF parasites, plates were incubated for 72 hours prior to addition of alamar blue reagent, and then incubated for another 48 hours. When BSF *T*. *congolense* were assayed, the BSF *T*. *b*. *brucei* protocol was used, with an initial density of 2.5 × 10^5^ cells/mL.

### Isobologram assays

To investigate drug-drug interactions, isobologram analyses were carried out using a fixed-ratio protocol previously described [37]. This assay uses the same principles as an alamar blue assay to test for cell viability over a range of drug concentrations but testing two drugs simultaneously.

For both drugs, the top concentrations used were chosen for the EC_50_ to fall near the midpoint of a 12-part two-fold dilution series. We typically started with 16× EC_50_ and fixed ratio solutions of drugs were made as follows: 10:0, 9:1, 8:2, 7:3, 6:4, 5:5, 4:6, 3:7, 2:8, 1:9, 0:10. These stocks were then added to the first column of 3 solid white flat-bottomed 96-well plates in duplicate. The compounds were then serially diluted 1:1 with the final well of each row left blank as a negative control. Finally, cells were added at a final concentration of 2 × 10^4^ cells/mL. Plates were then incubated for 48 hours and alamar blue reagent added as described above. The plates were read after another 24 hour incubation on a BMG FLUOstar OPTIMA microplate reader (BMG Labtech GmbH, Germany) as described above.

For each fixed ratio, an EC_50_ value was generated for either drug. These values were then used to obtain fractional inhibitory concentration (FICs) indices, which are defined as the EC_50_ values of drug in combination, divided by the EC_50_ values of those drugs acting alone [61]. The isobologram was then generated by plotting the FICs of one drug against the other.

The overall mean ΣFIC was calculated for each combination by adding the values of each individual FIC of either drug, and a mean ΣFIC value was used to assess whether the drug interactions were synergistic, indifferent or antagonistic.

### Metabolomics, LC-MS & data analysis

Samples for metabolomics analysis were acquired by rapidly quenching 8 × 10^7^ cells in log phase in a dry-ice/ethanol bath, to ~4°C. For each sample group, four replicates were grown independently. In experiments involving stable isotope labelling, three replicates were used and isotope-labelled compounds ([U-^13^C]-L-methionine, enrichment 99%, Cambridge Isotope Laboratory Inc., cat: CLM-893-H-0.1) were added at the moment the time course was initiated. After quenching, samples were centrifuged for 10 minutes at 1,500 × *g*, 4°C, and all experimental steps hereafter were done on ice to keep the samples cold. Subsequently, 5 μL supernatant was transferred to an Eppendorf containing 200 μL extraction solvent (Appendix D), and the rest removed. Cells were re-suspended in the remaining supernatant and transferred to an Eppendorf so that they could be centrifuged at 1,500 × *g* for another 5 minutes. Remaining supernatant was carefully removed, and the cells re-suspended in 200 μL extraction solvent. All samples, including a blank and fresh medium control, were then left in a shaker at 4°C for one hour. Subsequently, samples were spun down at 16,060 × *g* for 10 minutes, and the supernatant was transferred to a 2 mL screw-top tube. From each sample, 10 μL was transferred to an empty tube to produce a quality control sample. Finally, air in the tubes was displaced with argon gas to prevent oxidation of metabolites, and samples were stored at -80°C until they were analysed by liquid chromatography-mass spectrometry.

All mass spectrometry of metabolite samples was carried out by Glasgow Polyomics. Metabolomics samples were separated by hydrophilic interaction liquid chromatography (HPLC) using a ZIC-pHILIC (polymer-based HPLC) column (Merck). Two solvents were used in the column. Solvent A was 20 mM ammonium carbonate in H_2_O and solvent B was 100% acetonitrile. Mass detection was carried out using an Exactive Orbitrap mass spectrometer (Thermo). The mass spectrometer was run in positive and negative mode with an injection volume of 10 μL and a flow rate of 100 μL/minute.

### Preparation of slides & microscopy

Mitochondria were stained using Mitotracker Red (Invitrogen) prior to fixation and mounting. To achieve this, cells (typically 1 mL at 5 x 10^5^ cells/mL) were incubated for 5 minutes at 37°C, 5% CO_2_, with a final concentration of 100 nM Mitotracker. Upon completion, cells were centrifuged for 5 minutes at 1,500 × *g* and washed twice in fresh medium before cells were fixed.

Trypanosomes were grown to mid-log phase before control and treatment cultures were started at a density of 2 × 10^5^ cells/mL. For each time-point, at least 3 mL culture was centrifuged at 1,500 × *g*, washed twice with sterile 1× PBS, and finally re-suspended in 500 μL PBS. Samples were fixed by adding a final concentration of 2% formaldehyde, and incubated for 15 minutes at room temperature. Subsequent to a wash with PBS, cells were transferred onto a poly-L-lysine-coated slide, and left to air-dry in a safety cabinet. Dried slides were rehydrated and washed in PBS, and a counterstain consisting of 1× PBS with 3 μM 4,6-diamidino-2-phenylindole (DAPI) was applied to the slide, before mounting with a coverslip that was sealed with clear nail varnish. Slides were analysed with a Zeiss axioscope (Scope.A1, Zeiss).

To ascertain whether compounds affected the *T*. *b*. *brucei* cell cycle, cells were prepared for microscopy analysis as described above. Cells were then counterstained with DAPI and classified according to the numbers of nuclei and kinetoplasts they had, as a direct correlation to phases of the cell cycle, as described in several publications [62, 63]. Cells in G1 phase have one nucleus and one kinetoplast (1N1K). Kinetoplast replication (S-phase) then initiates (1N2K) prior to nuclear division (2N2K). Finally, completion of the cell cycle leads to two cells in G1 phase.

To analyse any potential changes in cell cycle, >300 cells were counted in multiple samples and the number of cells in different cell cycle stages, as well as those in 2N1K and MNMK (‘M’ defined as ‘multiple’ organelles) phase, were calculated as percentages of the total number of counted cells.

To measure the distance between nucleus and kinetoplast, images were obtained from DAPI-stained samples and these imported into the Fiji software [64]. Distances were measured after the scale was set using the “measure” tool. For each sample group, 30 measurements were taken from three independent microscopy experiments.

### Computational methods

Graphical representation of data was created using the Graphpad Prism software (v6.0, GraphPad Software, www.graphpad.com), R [65] or Inkscape. Statistical analyses were carried out using Graphpad Prism, SPSS, Microsoft Excel and R. Further computational analysis was carried out using R. Analysis of microscopy images, as well as processing of blots, was carried out using Fiji [64].

Raw data produced by the mass spectrometer were converted to the mzXML format using msconvert [66]. This step also split the polarity of the data. Files were then converted to peakML files with XCMS, which uses the Centwave function to pick peaks, converting every individual file to the peakML output.

Metabolite identification was done using Ideom [67, 68]. Where targeted metabolomics using stable isotopes labelling was carried out, mzMatch-ISO [69] was used for metabolite identification, and all hits were then confirmed using Ideom. Putatively identified metabolites were also analysed using the Metacyc [70] and Kegg [71] databases. Further metabolomics figures, including heat maps and PCA plots, were either created using Metaboanalyst [72], Microsoft Excel, R or GraphPad Prism.

## Supporting information

S1 Table Metabolomics dataset for AN5568 and sinefungin treatment of *T*. *b*. *brucei* Lister 427. (XLSX)

S2 Table Metabolomics dataset for AN5568 and DTT treatment of *T*. *b*. *brucei* Lister 427. (XLSX)

S3 Table Genes containing MTase domains identified in *T*. *b*. *brucei*. (XLSX)

S1 Fig Tracing 13C distribution in AN5568-treated cells: Lysine metabolism. Methylated lysine contains methyl groups originating from L-methionine. Whilst lysine methylation has been shown to be involved in the generation of L-carnitine, no 13C-labeled L-carnitine was detected in wild-type or AN5568-treated cells.
(TIFF)

S2 Fig AN5568 and sinefungin sensitivity in methyltransferase overexpression lines. Six T. brucei overexpression lines were generated and their sensitivity to both AN5568 and sinefungin was tested. Whilst no methyltransferase conferred resistance to AN5568 when overexpressed, two overexpressors did show a moderate increase in sinefungin resistance (* = *P*<0.05, Student’s *t*-test).
(TIFF)
