# Supplementary figures and images for: Benzoxaborole treatment perturbs S-adenosyl-L-methionine metabolism in Trypanosoma brucei
Source: PLoS Negl Trop Dis. 2018 May 14;12(5):e0006450. doi: 10.1371/journal.pntd.0006450 (PMC5976210; doi:10.1371/journal.pntd.0006450)

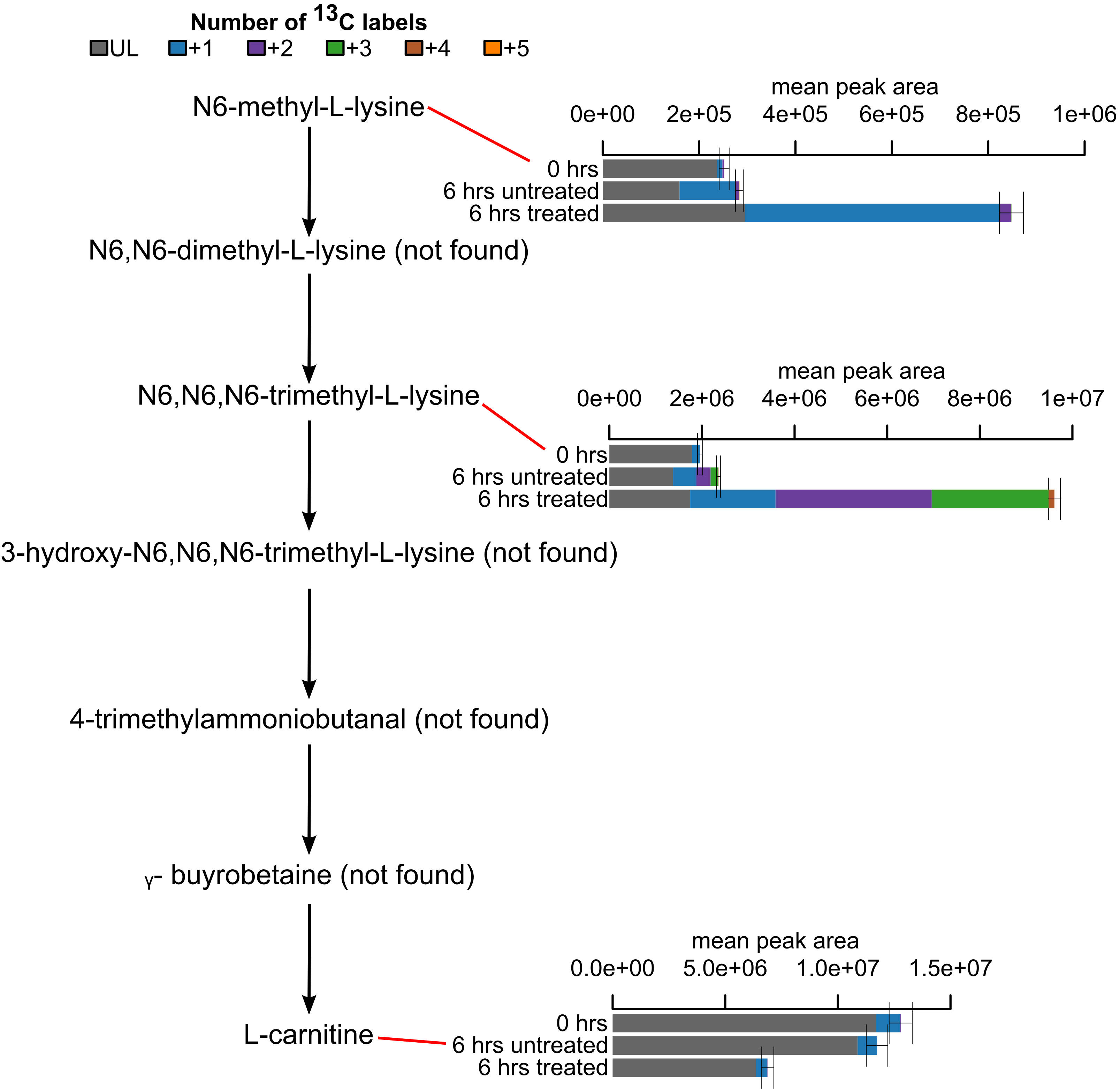

Supplement: S1 Fig — Methylated lysine contains methyl groups originating from L-methionine. Whilst lysine methylation has been shown to be involved in the generation of L-carnitine, no 13C-labeled L-carnitine was detected in wild-type or AN5568-treated cells. (TIFF) [file pntd.0006450.s004.tiff]

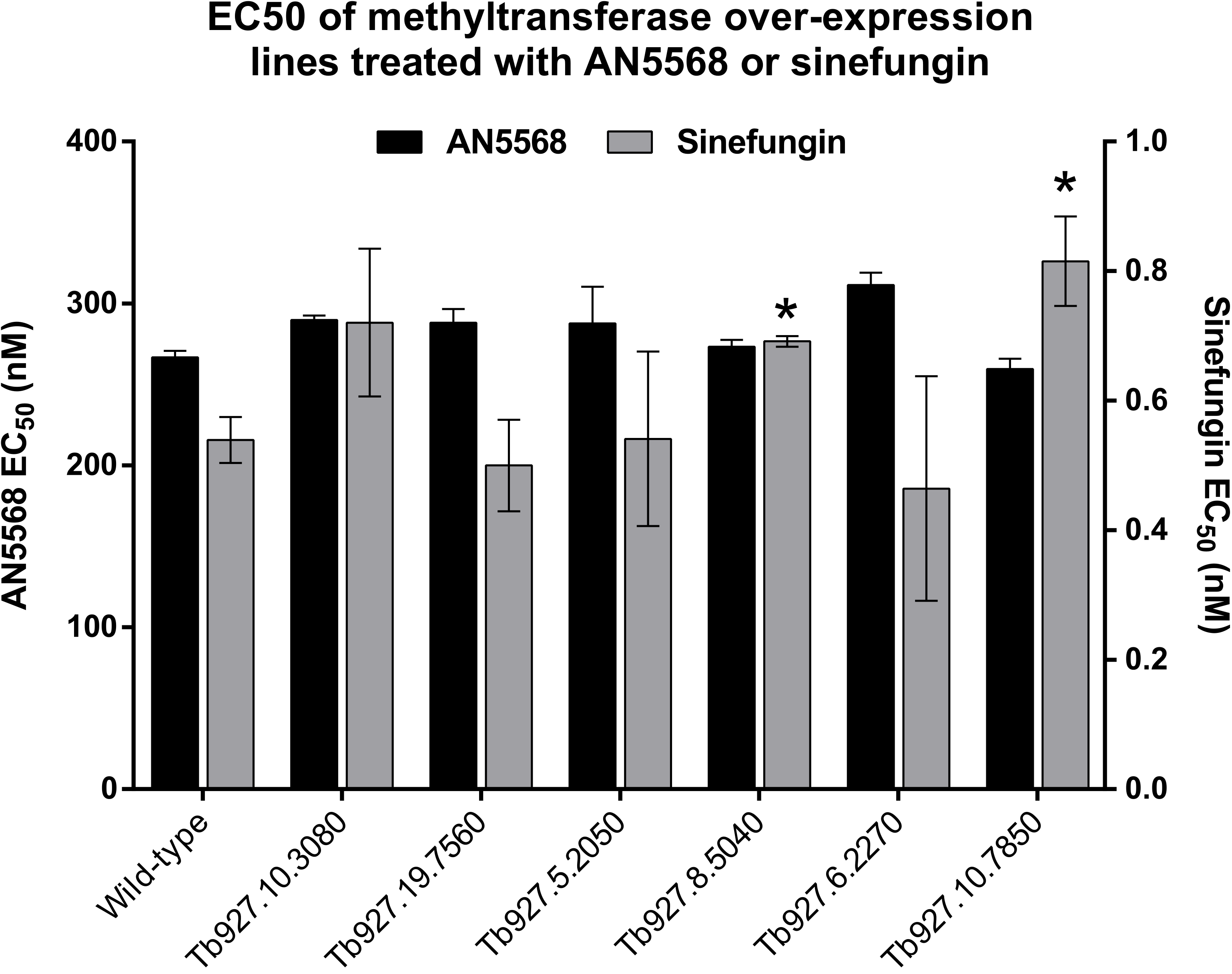

Supplement: S2 Fig — Six T. brucei overexpression lines were generated and their sensitivity to both AN5568 and sinefungin was tested. Whilst no methyltransferase conferred resistance to AN5568 when overexpressed, two overexpressors did show a moderate increase in sinefungin resistance (* = P<0.05, Student’s t-test). (TIFF) [file pntd.0006450.s005.tiff]
